# Holistic Approach in Designing the Personalized Bone Scaffold: The Case of Reconstruction of Large Missing Piece of Mandible Caused by Congenital Anatomic Anomaly

**DOI:** 10.1155/2020/6689961

**Published:** 2020-11-22

**Authors:** Jelena R. Milovanovic, Milos S. Stojkovic, Karim N. Husain, Nikola D. Korunovic, Jovan Arandjelovic

**Affiliations:** ^1^Department of Production Information Technologies, University of Nis, Faculty of Mechanical Engineering, 18000 Nis, Serbia; ^2^University of Al-Qadisiyah, Faculty of Mechanical Engineering, 58001 Al Diwaniyah, Iraq

## Abstract

The paper reports on the importance of applying the holistic approach in designing a *personalized bone scaffold*, but also all other kinds of personalized implants. In addition, the paper attempts to point out the important aspects of the design of a PBS against which the quality of a realistic and applicable design solution should be assessed. The holistic approach refers to the adaptation of design features of a bone scaffold to the multilateral specifics related to the particular patient, its surgical case, and curing treatment. To ensure a successful application, five aspects of personalized bone scaffold design should be considered while it is being adapted: anatomical congruency, mechanical conformity, biochemical compatibility and biodegradability, manufacturability, and implantability. To demonstrate the importance of applying a holistic approach in designing a personalized bone scaffold, the paper shows a case where a patient-specific scaffold aimed at the reconstruction of a *large missing piece of mandible* was designed. The research resulted in a series of recommendations regarding the methods of bone geometry reconstruction and scaffold design. The paper sheds new light on the desired mechanical properties of a personalized bone scaffold while also recommending possible design parameters for optimizing the construction according to these properties. Finally, it recommends a possible procedure of integral production of personalized bone scaffold and bone graft. The presented so-called holistic approach announces a new systematic process of designing a personalized bone scaffold, which, although requiring a comprehensive consideration of complex requirements, is inevitable to make the designed solution applicable.

## 1. Introduction

To improve the process of recovery of damaged tissue, the current research in the field of tissue engineering is focused on developing the tissue substituent that is designed for each patient individually, the so-called personalized or patient-specific solution. With regard to the bone scaffold creation, which is an integral part of the bone tissue substituent, primarily serving to provide mechanical support to the weakly consolidated granular consistency of the bone graft, *personalization* comprehends multilateral adaptation of a bone scaffold design: its geometry should be shaped in accordance with the patient anatomy and implantation conditions whereas mechanical and biochemical features of the scaffold structure should be harmonized to the corresponding patient's specifics. Typical cases when the use of p*ersonalized bone scaffold* (PBS) is indicated are related to the reconstruction of a bone whose structure is significantly damaged; that is, a large piece of a bone is missing. The term *large missing piece of the bone* refers to a bone piece so large that is hard to expect to be recovered by the nature itself. There are typical cases why some large pieces of the bone could lack. The first case refers to the bones that have been damaged by a mechanical injury (fractures) where broken parts of the bone cannot be placed and fixed in a natural position. A typical example is multiple bone fractures where small or large parts are dislocated, and some part(s) of the bone is/are lacked or crushed. The second case refers to a bone in which a large part is affected by a tumour. The third case refers to a bone which is affected by a congenital or developmental anatomical anomaly. A typical example is related to the requirement for limb bone lengthening for functional and aesthetic reasons. In any case, it is necessary to try to create and implant a permanent replacement for the missing part of the bone. In principle, there are two approaches: (1) replacing that part of the bone with endoprosthesis and (2) trying to induce the body to regenerate the missing part on its own. The second approach is certainly better, but it is not always possible. Age, that is, the regenerative capacity and general health of the patient, the possibility of fixation, and mechanical viability are factors that direct the determination of which these two approaches can be applied. The approach of inducing the body to recover on its own in cases where a large part of the bone is damaged can be aided by the implantation of a specific type of implant, the bone scaffold. The first goal of bone scaffold implantation is to hold the graft (proto tissue) and to provide the most efficient regeneration of the missing (bone) tissue volume. Regarding this goal, it is easy to recognize the need to optimize the geometry and mechanical properties of the scaffold according to the specifics that characterize a particular patient's case. The second goal comprehends thorough and fast growth of the bone tissue through the volume of the scaffold (missing piece), connecting with healthy nearby bone tissue without inducing the (bio)pathologic responses like infections. In the context of this goal, the scaffold bioactivity that could reflect in its time-controlled biodegradability and its capability to absorb and dose active substances according to the external inducing signal becomes one of its utmost important features [1].

## 2. Objectives and Challenges of PBS Application

The objectives and corresponding challenges related to the personalization of a human bone scaffold design can be identified by the analysis of the main features that PBS should possess.

### 2.1. Aspect of Anatomical Congruency

**Figure 1:**
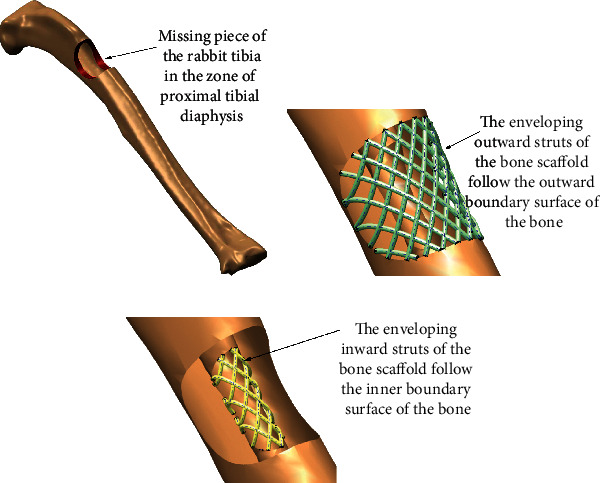
Anatomical congruency of the personalized bone scaffold.

The geometry of PBS should follow the enveloping boundary surfaces of the volume of the missing piece of the bone. Implicitly, this feature requires enveloping outward and inward struts of PBS to follow the boundary surfaces of the volume of the bone piece that should be substituted by PBS (Figure 1).

**Figure 2:**
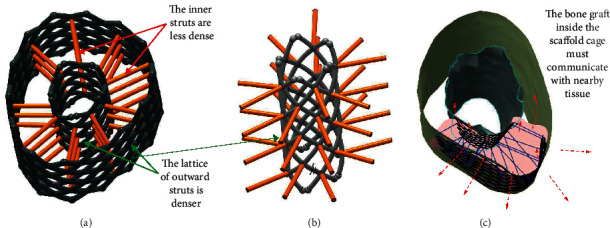
The lattice (cage) of inner cross-connecting and outward/inward enveloping struts.

To enable the generation of the callus that will preserve the anatomical shape of the bone as much as possible, as well as enabling transferring the load through the expected anatomically defined directions, the imperative for the design of the bone scaffold is to be congruent to the anatomic shape of the bone region where it should be implanted. In addition, it is essential to build the struts in such a manner to enable maximal communication with nearby tissues. The “tissue communication” facilitates the revascularization and reinnervation of the proto tissue of the bone graft, which is a precondition for thorough and fast bone tissue formation. This request suggests that PBS should be designed to be as hollow as possible. At the same time, PBS must hold the semiliquid structure of the bone graft within the cage of PBS in the first several weeks of the recovery, while the graft is being solidified and connected with other nearby tissues. These two design objectives, hollowness or airiness and closedness (needed to keep the graft within the volume of PBS under the load), are to some extent contradictory. The anatomically shaped cage-like or lattice-like structures of the PBS, where the outward struts are disposed in a bit denser manner than the inner struts, may be considered as an optimal solution [2, 3] (Figure 2).

**Figure 3:**
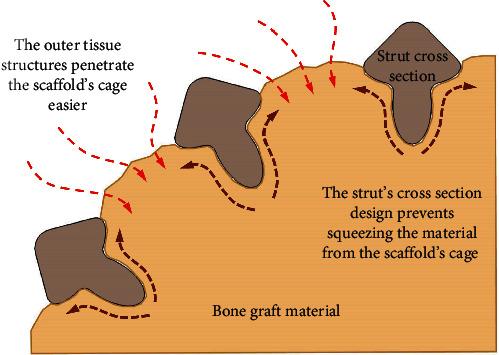
The cross section design of the PBS struts and its effects.

Additionally, proper design of the cross section of outward and inward struts could help for better holding the graft material inside the scaffold. The cross section of these struts could be designed to provide more resistance for the mass inside to get out of the cage than for the organic structures to penetrate the space of the cage (Figure 3).

The granules of autologous crushed bone (or granules of allogeneic or synthetic bone material) in the mixture of a bone graft serve as microcarriers for a soft material of the bone graft, which is usually consisting of fat tissue, stem and/or progenitor cells, and other soft and liquid substances. In a way, these granules consolidate the semiliquid material of the bone graft. At the same time, these granules play a role of a sort of generic centres for ossification process spreading.

### 2.2. Aspect of Mechanical Conformity

**Figure 4:**
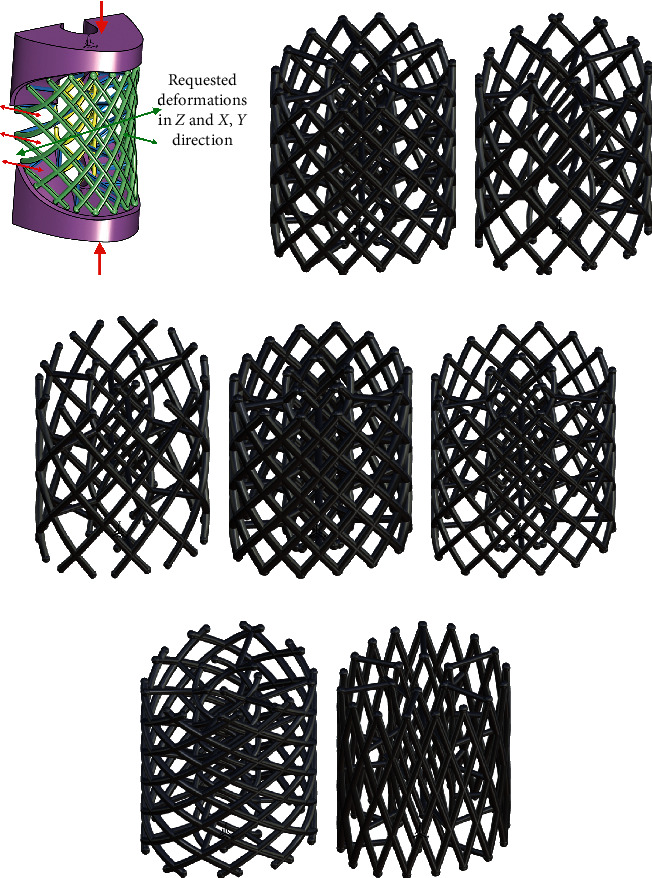
Design parameters that can be used for adjusting the requested deformation [2]. (b) Initial design. (c) Struts' density. (d) Low struts' density. (e) Thick cross section. (f) Thin cross section. (g) Wide strut's angle. (h) Sharp strut's angle.

The PBS should be designed in a way to enable the required magnitude of deformations in the specific directions under the most common load/constraint cases inherent to the patient [2, 4]. Actually, properly designed construction and material selection for PBS can provide a suitable strength of the structure that will enable the required magnitude of deformation and fatigue resistance (Figure 4). The speed and thoroughness of the ossification process are directly related to the mechanical stimulus that the traumatized bone region is exposed [5, 6]. Mechanical stimuli that are induced within the neighbouring traumatized tissue and within the prototissue of the bone graft while transferring a part of the mechanical load accelerate the ossification. The proper mechanical stimulus is in the function of magnitude and direction of bone graft deformation, that is, PBS deformation. That is why the PBS construction should be designed in a way to provide the proper deformations under the load cases usual for the particular patient (neither too small, neither too intensive mechanical stimulus) (Figure 4). At the same time, it should endure the necessary number of cycles of the load that will be imposed while the recovery process ongoing without losing its support function. There are a few research endeavours reporting on the optimization of lattice-like scaffold structure in terms of mechanical characteristics required for the specific patient [2, 4].

### 2.3. Aspect of Biochemical Compatibility and Biodegradability

**Figure 5:**
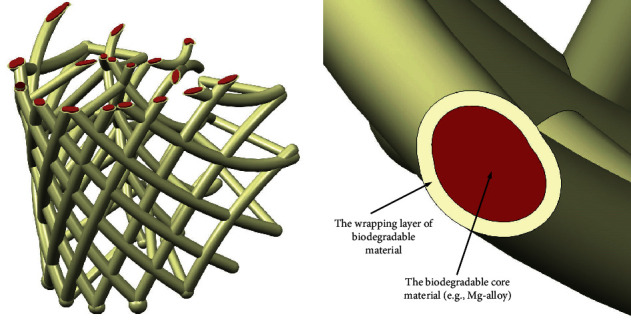
Multilayered material struts as a solution for time-controlled biodegradability.

In terms of biochemical properties, the material of PBS must not cause any kind of pathologic effects on the patient's organism. Furthermore, the ideal PBS material should be biodegradable, whereby dissolution products of the PBS must not be toxic to the patient's organism. The volume of the PBS struts that are being dissolved in time should be substituted by the native bone tissue, which is being generated from the proto tissue of the graft at the same time. The objective regarding biodegradability is to enable control of the biodegradation process in time. This would make controlling the load that PBS should carry during the recovery period possible. A solution that might be applicable is to wrap a core material of the scaffold struts (Figure 5). The type of wrapping layer material and its thickness may be used to control the period of its degradation [7]. After the period of wrapping material degradation, the core material of the struts is expected to start degrading also. Not less important than time biodegradability is the capacity of the PBS to react in accordance with the state of the tissue that is being recovered. The PBS should be able to contain biomedical, active substances in their struts that can be released in the space around it to prevent infection and stimulate ossification and cell differentiation. It could be especially valuable if the dosing of these bioactive substances may be controlled by some external induces like a specific magnetic field [1].

### 2.4. Aspect of Manufacturability

**Figure 6:**
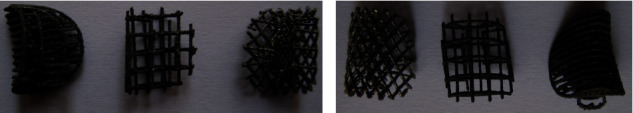
Different designs of the anatomically shaped lattice scaffold (truss) fabricated by the DMLS process [8, 9].

The PCB should be manufactured in the clinical sterile environment regularly. Considering the complexity of the PBS geometry (especially, if the bone graft should be built concurrently), it is no doubt that the PBS manufacturing can be done efficiently only by an *additive manufacturing technology* (AMT). However, no current AMT is quite suitable for the manufacturing of the hereinbefore described PBS. Due to some limitations, each of the currently available AMTs is deficient in some sense [8, 9]. Although SLS, SLM [4], DMLS, and EBM can create the complex 3D lattice that is wanted (Figure 6), the current fabrication process of these kinds cannot sinter the biodegradable material, neither more materials concurrently.

**Figure 7:**
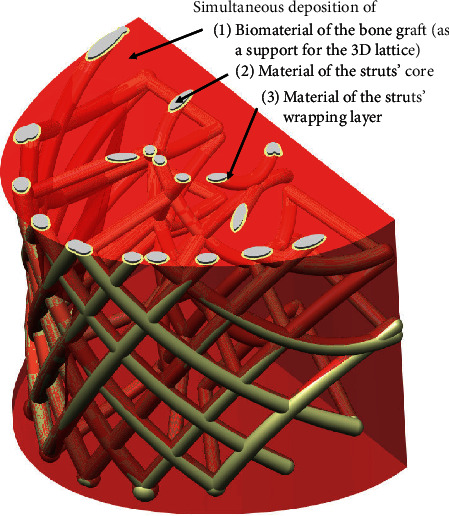
The PBS and its bone graft in the fabrication process.

Probably, the most promising current AMT technology is FDM for two reasons: (1) it may be easily adapted to deposit biocompatible, biodegradable materials, and even the bone graft mixture and (2) it is relatively cheap regarding any other AM technologies. The low accuracy of this kind of technology does not have great importance, since the geometric and dimensional tolerances of the PBS and other orthopaedic implants are not so tight as for the parts related to conventional mechanical engineering [10–12]. The FDM machine should be able to deposit at least two biodegradable materials, the core of the PBS struts and a wrapping layer that are going to be used for controlling the degradation during recovery (Figure 5). In addition, along with the material for PBS struts, the syringes of that FDM machine should be able to deposit bone graft substances. The deposited bone graft biomaterial can support the strut's material, since it is expected to build the PBS in which geometry is not a simple multilayered two-dimensional pattern of fibres [13] (Figure 7).

### 2.5. Aspect of Implantability

**Figure 8:**
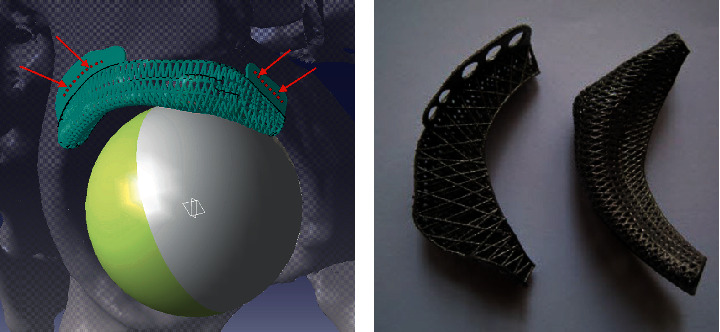
Adaptation of a personalized titanium scaffold design aimed for the reconstruction of a crushed acetabulum labrum may be seen in adding the fastening latches for fixing screws placement: (a) The scaffold lips are added to facilitate fastening and (b) Ti-alloy scaffold fabricated by DMLS.

The PBS design should be characterized by suitable fastening elements that facilitate implantation, making it fast and preventing the scaffold mispositioning. The fastening elements (attachment) are expected to be designed in accordance with the patient's anatomy and the particular surgical operation plan (Figure 8).

**Figure 9:**
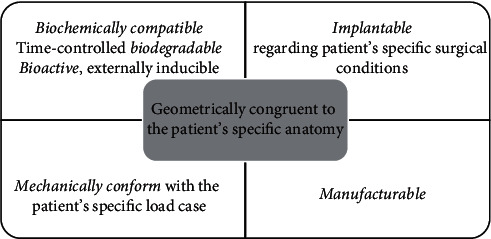
The main features of the PBS.

Hence, as it is explained hereinbefore, the PBS design needs to fulfill a complex of patient-specific requirements. It must be biochemically compatible with patient's organism, geometrically congruent to the patient's anatomy, biodegradable (time-controlled if possible), and preferably bioactive, mechanically tuned to produce the required deformation for the patient's specific load case, adjusted to the patient's specific implanting conditions and finally manufacturable, Figure 9.

The recovery process in its wholeness and not just separate objectives like anatomical congruency, biocompatibility, mechanical conformity, easiness of implanting, or similar, should be the fundamental reference to be considered while adapting, that is, personalizing the bone scaffold.

## 3. Related Research

In most of the papers, which are related to the topic of personalized human bone implants, the personalization is about the adaptation of the geometry of endoprostheses [14] or fixation elements (plates, [15]) to the geometry of the injured bone. There are many papers reporting on conducted analyses and simulations of mechanical behaviour of the bone [16] or corresponding endoprosthesis and fixation elements or their assembly [17] performed by the finite element method. However, there is almost no reported research in which a FEM analysis is performed to adapt the geometry of the implant structure to provide targeted deformations that would affect tissue recovery, i.e., stimulating the ossification process.

There are also reports on design solutions of personalized endoprostheses and fixation elements, which consider implantation and production aspects [18]. The advent of additive technologies enabled more intensive research related to the design and manufacturing of personalized implants. There are numerous papers in which special attention is paid to the application and benefits of using additive technologies for the manufacture of personalized endoprostheses [19]. Regarding the procedure and strategy of making personalized implants, there are several papers and patents [20, 21]. However, in these materials, one can find just a few recommendations for the target parameters of design adaptation as well as for the assessment of the quality of a design solution.

When the field of research is narrowed down to personalized scaffolds, it is very difficult to find a paper reporting on research related to the design of personalized bone scaffolds, and even fewer in which the bone scaffold design has been perceived in a comprehensive way. If one were to try to group research in this domain, it could be said that there are four basic subdomains of research. In the first subdomain, the focus is on scaffold geometry, most often with special emphasis on the imitation of bone tissue microstructure [22]. This research is dedicated to the modelling and optimization of the porous structure of a bone scaffold [23]; however, the design solutions resulting from these studies are not related to the comprehensive scaffold personalization. The focus of a second subdomain is on the analysis and simulation of mechanical properties of various types of scaffolds [24]. The third subdomain is dedicated to testing of additive technologies capabilities for bone scaffolds production [25–27]. Finally, there are scaffold design studies that include a combination of the previous three subdomains [4]. However, there are just a few recent papers [28] showing the design solutions that encompass all essential design aspects necessary to make PBS successfully applicable. Moreover, there is a lack of proposals for a reference framework that would provide guidelines for perceiving the PBS design solution comprehensively as well as for its applicability assessment. With this paper, we attempt to provide an initial step towards this framework by proposing a set of criteria for comprehensive applicability assessment of PBS design.

## 4. Materials and Methods

**Figure 10:**
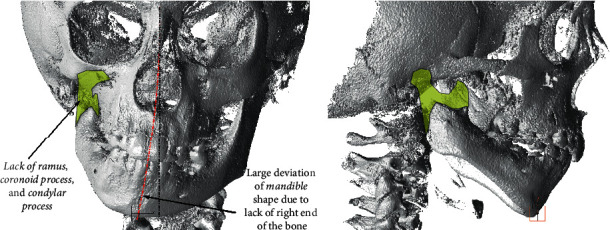
The case for working out the holistic approach in designing PBS and personalized implant.

The example we took as a reference example to test the holistic approach was the preadult human mandible that lacks a large part due to a congenital anatomical anomaly (Figure 10). Moreover, the bone is largely deformed due to lack of a part of the bone. The half of the right ramus and complete coronoid and condylar processes including the mandible condyles remained undeveloped (Figure 10).

Due to the absence of temporomandibular joint and muscles attachment areas, the stump on the right side of the mandible became asymmetrical with respect to the corresponding part of the left side.

The aim was to design and produce a PBS that could be implanted providing the temporary mechanical support to the bone graft material inserted into the cage of PBS. The geometry of the lattice-like scaffold, which is expected to be implanted together with an appropriate bone graft to substitute the missing part(s) of the mandible, should provide the targeted anisotropy of the mechanical properties in terms of its stiffness in the specific directions. Since this is a bone of a preadult patient, a boy who is facing a period of intensive growth of all tissues including bone tissue as well, a big capacity for ossification is considered an opportunity to implant the scaffold and graft on time. Instead of the usual treatment that recommends waiting for the bone tissue to stop growing (and get its final shape) and only then to perform subsequent extension operations, the application of PBS with a bone graft allows alternative treatment, to implant the bone substitute before the tissue growth. In this way, it is possible to utilize the ossification capacity of a young growing bone for the natural growth of a large bone mass that is missing. This treatment could prevent the bone from suffering additional deformation during the intensive growth caused by forces generated by surrounding muscles and bones. The missing part of the bone could be formed before extensive tissue growth, so it could follow the growth of other surrounding tissues correcting the existing anatomical anomaly. For the creation of mandible PBS, the method of holistic personalization has been applied, which took into consideration all the PBS design aspects that were described earlier in the paper.

## 5. Results

### 5.1. Digital Reconstruction of a Bone's Geometry

**Figure 11:**
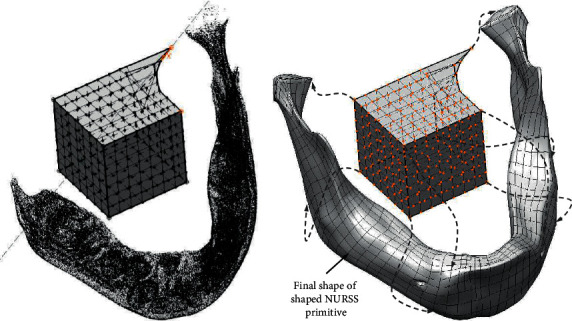
Creating the geometry of the bone by shaping Nonuniform Recursive Subdivision Surface primitive.

The inevitable step in making PBS is to build a geometric model of a (damaged) bone in the form in which it was before the damage. The most efficient way to try to shape a biological form, such as the boundary surface of а bone, characterized by complex topology is to use a surface subdivision modelling technique. Surface subdivision technique enables efficient shaping of a NURSS (Nonuniform Recursive Subdivision Surface) primitive in accordance with a target bone geometry (Figure 11), preventing the creation of cracks, voids, and wrinkles [11, 12, 29].

**Figure 12:**
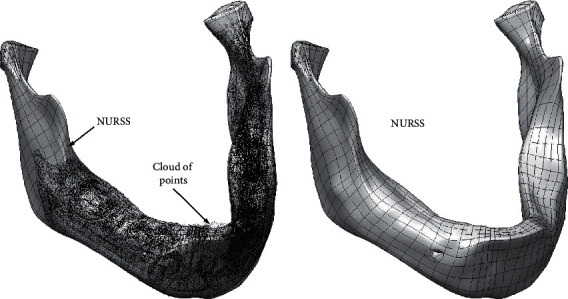
Mandible geometry reconstructed by regular NURSS mesh of nodes, edges, and faces.

The network of nodes and edges generated during the formation of the initial NURSS primitive is characterized by a regular spatial arrangement of nodes (Figure 12).

Near local deviations in surface curvature (e.g., bulges or dents on the bone), it is possible to form a denser network of control points and faces, whose subsequent translations and rotations can effectively model these local deviations.

### 5.2. Scaffold Designing

**Figure 13:**
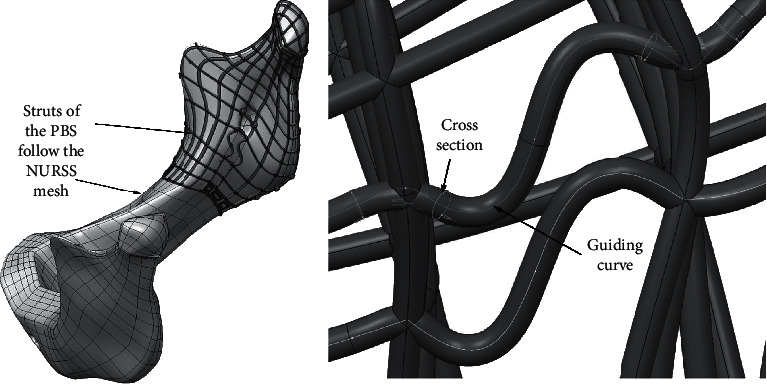
(a) PBS as an assembly of strut's UDFs laying over NURSS mesh. (b) A simple variant of a strut that enables larger deformation (to make the scaffold more elastic locally and in the specific direction).

The shaped NURSS mesh serves as a basis on which the struts of the scaffold can be arranged. PBS struts are being designed as groups of user-defined features (UDFs) whose main member is sweep protrusion form. It is characterized by a cross section and the guiding curve that shapes the protrusion line (Figure 13). The form of the cross section of the struts can be selected from a list of previously defined cross sections. The parameters that control the design of the cross section of the struts can be used later in adapting lattice structure design towards targeted mechanical properties by the FEM. Since each strut is a new instance of strut UDF, it is possible to make each strut of the scaffold unique in its shape, by fitting it to the desired mechanical characteristics.

### 5.3. Adaption of the Scaffold Design to the Required Mechanic Properties

To get the scaffold construction of the required structural strength for the period of tissue growth and recovery, a finite element optimization study has been conducted. The optimization parameters could be density (number) of struts, the shape and dimensions of the cross-sectional elements (Figure 4), the arrangement of appropriate (predefined) dilatation elements (struts).

The objective is targeted anisotropy in terms of mechanical properties, primarily flexural strength in the specific directions, and elasticity that will not obstruct the growth of proto tissue, neither its binding to surrounding healthy bone tissue. The elasticity of the PBS should be harmonized with the elasticity of the healthy part of the mandible to minimize the deformation difference between bone and PBS. Furthermore, the flexural strength of the PBS construction should be harmonized with the other (healthy) part of the bone.

The elongation capacity can be adjusted by inserting the so-called *polarized dilatation elements* of PBS instead of the usual struts (Figure 13(b)).

**Figure 14:**
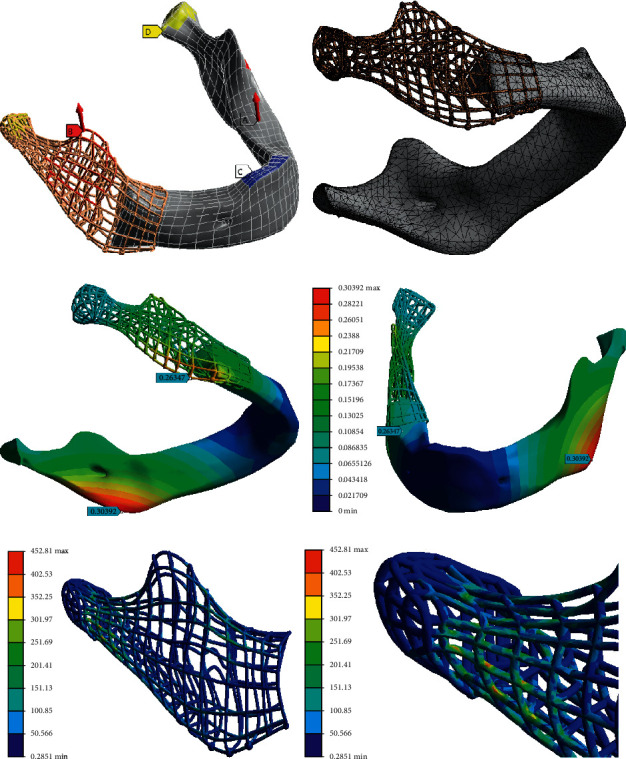
The FEA and design optimization regarding required mechanical properties. (a) Boundary conditions corresponding to anterior incisor bite. (b) Finite element mesh. (c) and (d) Deformation of the mandible and PBS assembly. (e) and (f) The field of equivalent stress in the PBS struts.

Due to the expected high concentration of stress in the zone between temporomandibular joint and ramus based on FEA (Figure 14), the lattice of the scaffold in the region of the condylar process is denser.

### 5.4. Adaption of PBS Design to the Implantation Conditions

**Figure 15:**
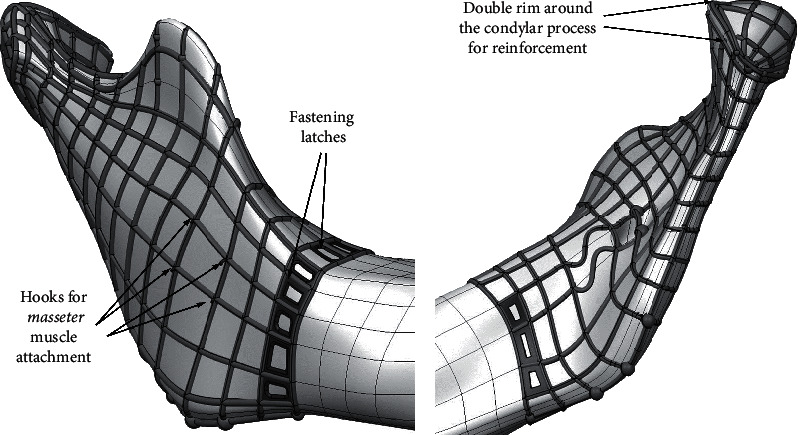
Personalized design elements for facilitation and improving the implantation.

When it comes to implantability, it is necessary to consider the following: (1) connection of the implant with the existing bone and method of attachment, (2) connection of implants with muscle tissue and connective tissue to ligaments, (3) bearing of the implant if it replaces the part of the bone that is a part of the joint surface of the bone, and (4) the method of inserting the implant into the body. Implanting elements have been added to the scaffold construction (Figure 15). Part of the scaffold at the interface with the bone stump is widened so that it can be elastically attached to the bone stump. Fastening latches have been added at selected places to utilize the placement of screws. In accordance with the surgeon's request, for the attachment of the masseter muscle with the scaffold and the bone graft inside, a kind of hooks were added to the basic construction of the scaffold on the ramus and the coronoid process. A specific cage of struts around the pivot zone of the temporomandibular joint was built to provide required functions during recovery.

Finally, the aspect of the installation activity itself must always be considered separately and in detail, so personalization of the scaffold solution is inevitable in this regard.

### 5.5. Manufacturing PBS

For the purposes of the experiment, the manufacturing process was designed to test the possibility of an integrated deposition of scaffold and bone graft material. This concurrent deposition of different materials indicates two additional aspects of manufacturing of PBS:

1. Integrated implants (assembly of scaffold and bone graft) must be manufactured in the clinical conditions right before the surgical operation or during the operation itself
2. That necessity consequently requires a specific kind of manufacturing system aimed for personalized implants design and production, which should operate in clinical conditions

Since, at the time of the experiment of manufacturing the integral personalized implant, there was no available AMT machine capable of stacking biological and scaffolds materials concurrently, it was decided to simulate the production process of deposition of more materials per layer using an FDM machine as a role model for some future systems.

**Figure 16:**
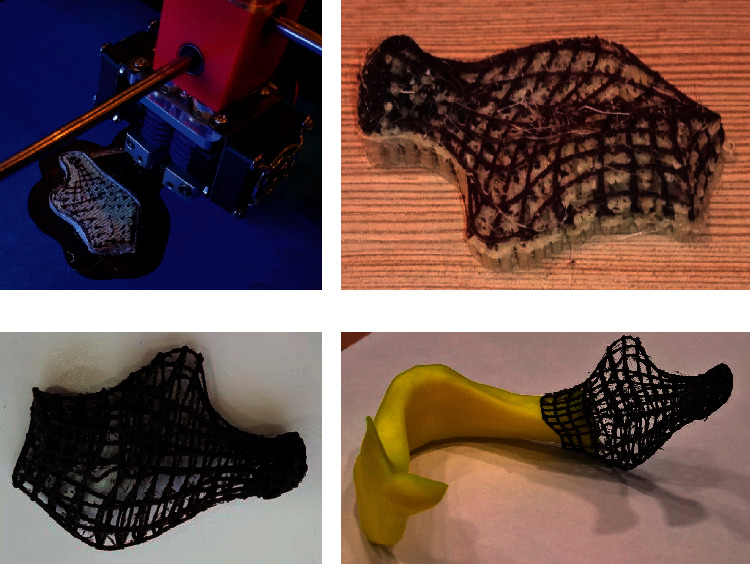
Manufacturing and mounting of PBS. (a) Stacking the material of the scaffold and its inner support structure. (b) Manufactured scaffold with its support structure. (c) The scaffold after the support structure was dissolved. (d) The scaffold, mounted on the affected mandible model as it should be during the surgical operation.

The FDM machine: CreatBot DX Plus was equipped with two extrusion heads, with the first head stacking the scaffold structure material (biopolymer) and the second head stacking the water-soluble support structure that played the role of biological material (graft) in the experiment (Figure 16).

### 5.6. Biochemical Compatibility and Biodegradability

Due to the difficulties of procuring the appropriate biodegradable material that should be able to apply within FDM process (i.e., for the FDM machines we had at our disposal), the elaboration of the biochemical compatibility and biodegradability aspects of the mandibular PBS solution was missed. However, in any future elaboration of PBS, this aspect must be considered.

## 6. Discussion

Although we knew that we would not be able to complete the research fast by implanting the mandible scaffold due to the long period needed to overcome the ethical obstacles of applying a human experimental implant, this PBS design study was conducted to show the need and importance of holistic approach when designing PBS and implants. The personalization of a bone scaffold implies adjustment of the geometry and structure multilaterally. That is why the conducted study included the following: (1) exploration of methods for efficient reconstruction of bone geometry, (2) working out the design of an appropriate PBS, (3) adapting the PBS structure to the requirements in terms of mechanical properties and implantation, and (4) working out of a potential production process of an *integral implant* made of scaffold and bone graft.

Personalization of a bone scaffold outweighs the need for scaffold geometry to be like a patient's specific bone anatomy. The optimization of the mechanical properties of the scaffold should not only ensure the load transfer but also provide the necessary deformation for the appropriate distinctive load cases, which is necessary for a strong stimulation of ossification. The design of the PBS should include structural elements that would facilitate or improve the process of incorporation into the patient's body as well as the attachment of the scaffold to healthy bone and muscle tissue. Finally, the study of a PBS development indicates the need for the so-called integral implants. Such implants should be made in clinical conditions where, in addition to the biodegradable material intended for scaffold struts building up, the necessary biological prototissue material would be stacked too. Holistic analysis of the role and implantation of PBS dispels some important misconceptions; for example, the geometry of the scaffold should mimic the geometry of the tissue. This seems completely unnecessary because the bone graft (especially the allograft, if it is possible to provide) is a part of an integral bone implant that should generate tissue geometry. During its transformation from prototissue into mature bone tissue, it forms an optimal and original bone tissue structure, just the one that specific patient needs. The bone scaffold is another part of that integral bone implant which should only temporarily ensure holding of the graft until it becomes mature bone tissue and connects to the other parts. The scaffold should be designed as a dynamic structure that should enable the growth of the original bone tissue through its volume, but also to provide appropriate stimuli to the surrounding tissue in the period until it resorbs.

### 6.1. Future Research

The presented study introduced a series of ideas related to the design of personalized bone scaffold whose effects have yet to be explored and evaluated: (1) conceptual design solution of lattice-like (or cage-like) scaffolds that are designed to hold bone graft and do not mimic the geometry of bone tissue, (2) the shape of the cross sections of the struts of the scaffold that provide a better grip of the graft inside the cage of the scaffold and facilitate the penetration of tissue from the outside to the inside of the scaffold, (3) deformable struts whose shape, orientation, and arrangement can be used to control the elasticity of the scaffold in different directions [2, 4, 6], (4) the attachment elements that should be used for attaching the scaffold to the surrounding bone tissue and muscles, (5) design of time-controlled biodegradable scaffold struts, (6) design of bioactive materials that can absorb and disperse bioactive substances in accordance with the external induces (e.g., external custom magnetic field like that reported in [1], and (7) method of production of integral implants that would enable using relatively cheap FDM process to fabricate the very complex 3D structure of a personalized bone scaffold.

## 7. Conclusions

The study of personalized scaffold design solutions aimed at the reconstruction of a large missing piece of mandible provided a more detailed understanding of the challenges in the personalization of a bone scaffold. Furthermore, the study has offered an initial framework of criteria for assessing the realistic and successful applicability of a PBS design solution. Of course, these criteria can be further elaborated and described in more detail, and we hope this paper will poke similar research in the near future. Finally, it seems that the offered holistic approach opens a new niche for further bioengineering research in the domain of patient-specific or personalized or customized bone implants and scaffolds.

## Data Availability

The data used to support the findings of this study are available from the corresponding author upon request.

## References

[1] Russo T., D’Amora U., Gloria A. (2013). Systematic analysis of injectable materials and 3D rapid prototyped magnetic scaffolds: from CNS applications to soft and hard tissue repair/regeneration. *Procedia Engineering*.

[2] Stojkovic М., Korunovic N., Trajanovic M., Milovanovic J., Trifunovic M., Vitkovic N. Design study of anatomically shaped latticed scaffolds for the bone tissue recovery.

[3] Korunovic N., Trajanovic M., Stevanovic D. Material characteriyation issues in FEA of long bones.

[4] Maietta S., Gloria A., Improta G., Richetta M., Santis R. D., Martorelli M. (2019). A further analysis on Ti6Al4V lattice structures manufactured by selective laser melting. *Journal of Healthcare Engineering*.

[5] Rosa N., Simoes R., Magalhães F. D., Marques A. T. (2015). From mechanical stimulus to bone formation: a review. *Medical Engineering & Physics*.

[6] Ghiasi M. S., Chen J., Vaziri A., Rodriguez E. K., Nazarian A. (2017). Bone fracture healing in mechanobiological modeling: a review of principles and methods. *Bone Reports*.

[7] Yongpei H., Qin Z., Renchuan Y., Lingshuang W., Mingzhong L. (2012). The relationship between secondary structure and biodegradation behavior of silk fibroin scaffolds. *Advanced Materials Science*.

[8] Milovanovic J., Stojkovic M., Trajanović M. (2015). Applicability analysis of additive manufacturing processes in fabrication of anatomically shaped lattice scaffolds. *Facta Universitatis, Series: Mechanical Engineering*.

[9] Milovanović J. (2014). “Application of additive technologies in fabrication of anatomical custom made scaffolds for bone tissue reconstruction”.

[10] Stojkovic M., Trajanovic M., Vitkovic N. (2019). Personalized orthopedic surgery design challenge: human bone redesign method. *Procedia CIRP*.

[11] Vitković N., Stojkovic M., Majstorovic V., Trajanovic M., Milovanovic J. (2018). Novel design approach for the creation of 3D geometrical model of personalized bone scaffold. *CIRP Annals*.

[12] Stojkovic M., Veselinović M. M., Vitkovic N. (2018). Reverse modelling of human long bones using T-Splines—case of tibia. *Technical Gazette*.

[13] Yang Y., Wang G., Liang H. (2018). Additive manufacturing of bone scaffolds. *International Journal of Bioprinting*.

[14] Mohammed M. I., Fitzpatrick A., Gibson I. Customised design of a patient specific 3D printed whole mandible implant.

[15] Xiaozhong C. (2018). Parametric design of patient-specific fixation plates for distal femur fractures. *Proceedings of the Institution of Mechanical Engineers. Part H*.

[16] Caiti G., Dobbe J. G. G., Bervoets E. (2019). Biomechanical considerations in the design of patient-specific fixation plates for the distal radius. *Medical & Biological Engineering & Computing*.

[17] Yan L., Lim J. L., Lee J. W., Tia C. S. H., O’Neill G. K., Chong D. Y. R. (2020). Finite element analysis of bone and implant stresses for customized 3D-printed orthopaedic implants in fracture fixation. *Medical & Biological Engineering & Computing*.

[18] Merema B. J., Kraeima J., Ten Duis K. (2017). The design, production and clinical application of 3D patient-specific implants with drilling guides for acetabular surgery. *Injury*.

[19] Wang J., Min L., Lu M. (2019). Three-dimensional-printed custom-made hemipelvic endoprosthesis for primary malignancies involving acetabulum: the design solution and surgical techniques. *Journal of Orthopaedic Surgery and Research*.

[20] Gao C., Wang C., Jin H. (2018). Additive manufacturing technique-designed metallic porous implants for clinical application in orthopedics. *RSC Advances*.

[21] Afaque R. M., Enpeng W., Junlei H., Jan E., Xiaojun C. (2020). A review on computer-aided design and manufacturing of patient-specific maxillofacial implants. *Expert Review of Medical Devices*.

[22] Chabanon M. (2015). “Multiscale study of a perfusion bioreactor for bone tissue engineering”.

[23] Poh P. S. P., Valainis D., Bhattacharya K., Van Griensven M., Dondl P. (2019). Optimization of bone scaffold porosity distributions. *Scientific Reports*.

[24] Wieding J., Souffrant R., Mittelmeier W., Bader R. (2013). Finite element analysis on the biomechanical stability of open porous titanium scaffolds for large segmental bone defects under physiological load conditions. *Medical Engineering & Physics*.

[25] Habib F. N., Nikzad M., Masood S. H., Saifullah A. B. M. (2016). Design and development of scaffolds for tissue engineering using three-dimensional printing for bio-based applications. *3D Printing and Additive Manufacturing*.

[26] Leong K. F., Cheah C. M., Chua C. K. (2003). Solid freeform fabrication of three-dimensional scaffolds for engineering replacement tissues and organs. *Biomaterials*.

[27] Mota C., Puppi D., Chiellini F., Chiellini E. (2015). Additive manufacturing techniques for the production of tissue engineering constructs. *Journal of Tissue Engineering and Regenerative Medicine*.

[28] De Witte T.-M., Fratila-Apachitei L. E., Zadpoor A. A., Peppas N. A. (2018). Bone tissue engineering via growth factor delivery: from scaffolds to complex matrices. *Regenerative Biomaterials*.

[29] Husain K. (2020). “Anatomically shaped lattice scaffold for large mandible trauma”.

